# Quality of death certification in Colombia

**DOI:** 10.25100/cm.v49i1.3155

**Published:** 2018-03-30

**Authors:** Ricardo Cendales, Constanza Pardo

**Affiliations:** Grupo de Vigilancia Epidemiológica del Cáncer, Instituto Nacional de Cancerología, Bogotá, D.C., Colombia.

**Keywords:** Data collection, vital statistics, cause of death, developing countries, data quality, health information systems., Recolección de datos, estadísticas vitales, causas de muerte, países en desarrollo, calidad del dato, sistemas de información en salud.

## Abstract

**Objective:**

To evaluate the quality of the certification of general death and cancer in Colombia.

**Methods:**

Validity indicators were described for each province and the cities of Bogotá, Cali, Manizales, Pasto and Bucaramanga. A factorial analysis of principal components was carried out in order to identify non-obvious relationships.

**Results:**

Were analyzed 984,159 deaths, among them there were 164,542 deaths due to cancer. 93.7% of the general mortality was well certified. The predominant errors were signs, symptoms and ill-defined conditions. 92.8% of cancer mortality was well certified. The predominant errors were due to poorly defined cancer sites.

**Conclusions:**

Certification of quality indicators in Colombia has improved. Given the good performance of the quality indicators for certificating general death and cancer, it is considered that this is a valid input for the estimation of cancer incidences.

## Resumen

**Objetivo:**

Evaluar la calidad de la certificación de la muerte general y por cáncer en Colombia.

**Métodos:**

Se describieron indicadores de validez para cada departamento y las ciudades de Bogotá, Cali, Manizales, Pasto y Bucaramanga. Se realizó un análisis factorial de componentes principales con el fin de identificar relaciones no evidentes.

**Resultados:**

Se analizaron 984,159 defunciones, dentro de las cuales había 164,542 muertes por cáncer. El 93.7% de la mortalidad general estaba bien certificada. Los errores predominantes fueron signos, síntomas y afecciones mal definidas. El 92.8% de la mortalidad por cáncer estaba bien certificada. Los errores predominantes fueron cánceres de sitio mal definido.

**Conclusiones:**

Los indicadores de calidad de certificación en Colombia mejoraron. Ante el buen comportamiento de los indicadores de calidad de la certificación de la muerte general y por cáncer, se considera que ésta es un insumo válido para la estimación de incidencia de cáncer.

## Introduction

The measurement and monitoring of the quality of mortality information is a very important component in the evaluation of health information systems in each country ^1^. Mortality is a fundamental input for the analysis of situations in health, monitoring results of public health programs, and planning needs in health services ^2^; while also being an essential input for estimating incidents of cancer ^3^ ^,^ ^4^.

In Colombia, around 12% of the population is covered by a group of cancer population registries (RPC, for its acronym in Spanish): Cali, Pasto, Bucaramanga, Manizales and Barranquilla ^5^. The RPC information and the available national mortality data allow for national incidence estimates for cancer. On a first analysis, the National Cancer Institute (INC, for its acronym in Spanish) evaluated the quality of the certification of general mortality and cancer mortality as part of the validation process of the information sources ^6^. This report reviews the progress of the quality of the certification of general mortality and cancer based on the official information of the national vital statistics registry system of the National Administrative Department of Statistics (DANE, for its acronym in Spanish). This information is necessary for the estimation of cancer incidences 2007-2011 ^7^ and other types of analysis with mortality ^8^ ^-^ ^10^.

This publication presents the evaluation of the quality of the certification of death in Colombia during the period 2007-2011, according to the province of occurrence; it also presents the results for the capital district (Bogotá) and the four cities in which the cancer population registries operate.

## Materials and Methods

A descriptive study of the quality of mortality information was made from death certificates in Colombia. The results are presented disaggregated according to the province or city where the death occurred. For the provinces that have municipalities with RPC, the data of these municipalities is excluded to avoid redundancy. Thirty two provinces were included, the capital district (Bogotá) and the cities of Manizales, Pasto, Cali and Bucaramanga (which comprise the towns of Bucaramanga, Floridablanca, Girón and Piedecuesta) that have active cancer population registries, which are endorsed by the International Agency for Research in Cancer (IARC, for its acronym in English) ^11^, with a coverage of 12% of the population that represents the country. The source of information is the official DANE mortality database for the five-year period 2007-2011. 

### Statistical methods

The description of the quality of the certification was made through simple percentages. Each aspect related to the lack of quality in the information was considered only once; for example, if the death was not certified by a physician, but it also had another certification problem, this record was not counted twice but only once as a record that had faults in its certification quality. The results are presented in a logical order in such a way that those that appear in the first column of the tables correspond to the first reason of lack of quality; those that appear in the second column correspond to records that have other faults different from the first; and so on (Tables 1 and 2).

### Indicators for the general evaluation of information quality

#### The International Classification of Diseases (ICD-10) was used to group and codify the causes of death

The following indicators were constructed: deaths from cancer certified as primary non-established, from poorly specified sites, or as a consequence of metastatic tumors (C76-C80, C97); cardiovascular deaths of ill-defined etiology (I47.2, I49.0, I46, I50, I51.4, I51.5, I51.6, I51.9, I70.9); injuries of indeterminate intentionality (Y10-Y34, Y87.2); deaths that were recorded as signs, symptoms and ill-defined conditions (R00-R99); deaths without sex information; deaths that were not certified by physicians; and deaths that don’t have any cause of error in the certification.

#### Indicators in the evaluation of quality of information on cancer mortality

The following indicators were constructed: deaths from uterine cancer of unspecified site (C55); deaths from cancer of non-stated primary, from poorly specified sites, or as a consequence of a metastatic tumor without an established primary (C76-C80, C97); deaths from cancer without age information; cancer deaths that were not certified by a physician; and well-certified cancer deaths.

### Analysis plan

In order to detect a possible underlying relationship structure, a factorial analysis of the principal components was carried out, both for the analysis of the certification quality of general mortality and for the analysis of the certification of cancer mortality; the Kaiser-Meyer-Olkin statistic and the Bartlett sphericity test were used to identify if there was an underlying relationship structure ^12^. Those factors that did not fit with the proposed solution of major factors were excluded from the analysis; the number of factors was selected with the help of graphic analysis; in those cases in which it was considered appropriate, an orthogonal rotation was made using the analysis of principal components as the extraction method, and the varimax with Kaiser normalization as a rotation method based on graphic analysis.

## Results

In the evaluation of the quality of the certification of general death for Colombia, there were 984,159 deaths considered for the five-year period 2007-2011. For general mortality in the area of ​​influence of the population registries of cancer in Bucaramanga, Cali, Manizales and Pasto, 138,716 deaths were analyzed (14.1% of the national mortality). 

### Quality indicators of certification of death due to general causes

The national percentage of duly certified deaths was 93.7%. The indicators that most affected the quality of the certification were: signs, symptoms, ill-defined conditions (2.0%) and ill-defined cardiovascular deaths (1.9%). Ten provinces had a percentage of deaths without errors in the certification lower than 90.1%, and they represented only 6.6% of the deaths in the country. Vaupés was the province with the lowest indicators in certification, with 77.2%. The quality of the general certification for deaths without errors ranged from 92.3% to 96.4% for the cities of Cali, Manizales, Bucaramanga and Pasto. The city of Bucaramanga showed a lower proportion in its indicators with respect to the other cities (Table 1).

**Table 1:** General evaluation of the quality of the certification according to the place of occurrence of the death, Colombia, 2007-2011.

Geographic ordering	Deaths from cancer of non-stated primary	Ill-defined cardiovascular deaths	Injuries of undetermined intentionality	Signs, symptoms and ill-defined conditions	Deaths without sex information	Deaths without age information	Deaths that were not certified by a physician	Deaths without errors in certification	Total deaths
Amazonas*	0.4	1.1	2.8	7.2	0.0	0.0	7.3	81.2	848
Antioquia*	1.2	1.4	0.9	1.5	0.0	0.0	0.0	94.9	148,653
Arauca*	0.5	1.7	1.1	1.7	0.0	0.0	0.3	94.7	4,494
Atlántico*	0.9	2.0	0.5	1.9	0.0	0.0	0.0	94.6	46,182
Bogotá D.C.**	1.2	1.4	1.6	2.7	0.0	0.0	0.0	93.1	159,432
Bolivar*	1.2	2.4	0.8	2.9	0.0	0.0	0.4	92.3	29,089
Boyacá*	0.8	3.1	1.5	1.4	0.0	0.0	0.3	92.8	29,704
Caldas*	1.0	2.2	0.8	1.2	0.1	0.0	0.1	94.7	12,166
Manizales§	1.2	1.1	0.4	0.9	0.0	0.0	0.0	96.4	15,651
Caquetá*	1.0	2.2	3.2	3.6	0.0	0.0	0.2	89.7	8,121
Casanare*	1.2	2.9	3.5	2.8	0.0	0.0	0.3	89.4	4,799
Cauca*	0.8	1.8	1.5	3.6	0.0	0.0	3.9	88.4	22,658
Cesar*	0.9	2.3	1.0	2.7	0.0	0.0	0.2	92.9	17,480
Chocó*	0.6	1.8	3.0	4.9	0.0	0.0	2.1	87.6	5,381
Córdoba*	0.6	2.4	0.7	2.1	0.0	0.0	0.8	93.3	26,801
Cundinamarca*	0.8	2.6	1.4	3.0	0.0	0.0	0.0	92.1	43,956
Guainía*	0.0	2.3	1.4	1.4	0.0	0.0	7.6	87.3	353
Guajira*	0.5	1.8	1.0	2.2	0.1	0.0	0.8	93.6	7,227
Guaviare*	0.5	1.3	3.4	3.3	0.4	0.0	0.1	91.0	1,334
Huila*	1.1	1.8	1.5	2.1	0.0	0.0	0.2	93.3	25,036
Magdalena*	0.8	2.3	0.7	2.1	0.0	0.0	0.4	93.6	21,732
Meta*	0.9	1.3	1.2	1.3	0.0	0.0	0.0	95.1	20,611
Nariño*	0.6	2.4	1.9	3.8	0.0	0.0	0.9	90.4	16,746
Pasto¤	0.9	1.6	0.9	0.8	0.0	0.0	0.0	95.8	14,671
N. Santander*	1.0	4.7	0.9	2.5	0.0	0.0	0.2	90.8	31,768
Putumayo*	0.4	1.2	4.7	2.1	0.1	0.0	1.7	89.8	3,572
Quindío*	1.2	1.4	0.2	0.4	0.0	0.0	0.0	96.7	16,577
Risaralda*	1.2	1.4	0.6	1.3	0.0	0.0	0.0	95.5	27,529
San Andrés*	0.3	2.5	0.2	0.9	0.0	0.0	0.1	95.9	952
Santander*	0.8	3.5	1.4	2.9	0.1	0.0	1.1	90.2	17,838
Bucaramanga^†^	1.2	2.4	0.4	3.2	0.0	0.0	0.4	92.3	30,725
Sucre*	0.9	3.1	0.6	1.7	0.0	0.0	0.5	93.2	13,360
Tolima*	0.7	1.3	0.7	0.9	0.0	0.0	0.2	96.2	35,803
Valle*	0.9	1.6	1.0	2.0	0.0	0.0	0.2	94.3	44,184
Cali¤	1.1	1.2	0.7	0.7	0.0	0.0	0.0	96.4	77,669
Vaupés*	0.0	0.8	3.8	3.2	0.0	0.0	15.0	77.2	474
Vichada*	0.2	2.4	2.9	3.1	0.7	0.0	1.0	89.7	583
**Colombia**	1.0	1.9	1.1	2.0	0.0	0.0	0.3	93.7	984,159

‡ The geographical order of the country is defined in regions (two or more provinces),

* provinces (several municipalities), followed by special districts

**, § municipalities and metropolitan areas

† (two or more municipalities).

† Metropolitan area of Bucaramanga (Bucaramanga, Floridablanca, Girón, Piedecuesta)

**Figure 1:**
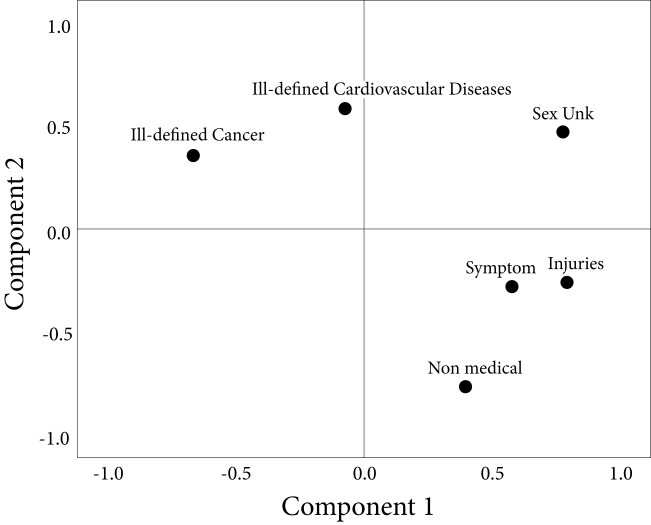
Results of the analysis of principal components in the general evaluation of the quality of the certification (rotated graph), Colombia, 2007-2011. Extraction method: Principal component analysis and Rotation method: Varimax standardization with Kayser.

The analysis of principal components showed two that can explain the lack of quality: the first one is related to the lack of certification by a physician and the consequent inadequate certification of death, either as a sign, symptom or ill-defined condition or as an undetermined intentionality injury (Amazonas, Cauca, Guainía, Vaupés); the second component has to do with the provinces in which, despite having certification by a physician, there are errors in the certification of cancer deaths and deaths due to cardiovascular causes (Santander, Norte de Santander, Boyacá, Sucre) (Table 1 and Fig. 1). 

### Quality indicators of cancer death certification

164,542 deaths were analyzed. The percentage of cancer deaths duly certified was 92.8%. The indicators that most affected the quality of the certification were deaths due to poorly defined cancer sites (6.1%) and deaths from ill-defined site uterine cancer (0.9%).

**Figure 2:**
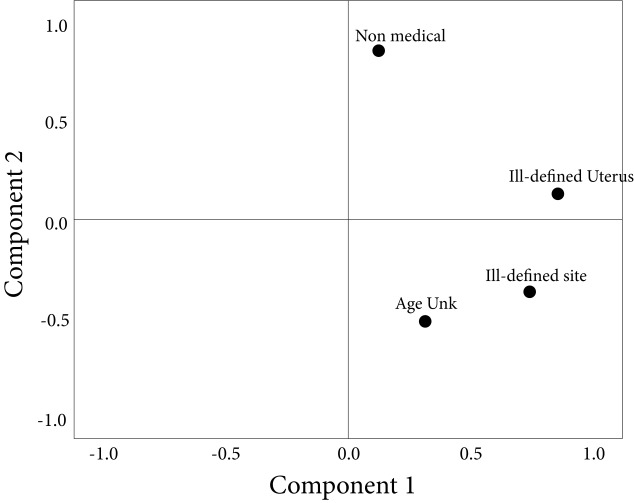
Results of the analysis of principal components in the evaluation of the quality of the certification by cancer (rotated graph), Colombia, 2007-2011. Extraction method: Principal component analysis and Rotation method: Varimax standardization with Kayser.

The analysis disaggregated by provinces showed 10 provinces with a percentage of deaths duly certified that were lower than 91.6%, which represented 9% of the total deaths due to cancer; Amazonas was the province that had the lowest indicators in the quality of cancer mortality certification (Table 2). In the analysis by cities, Bucaramanga, Cali, Manizales and Pasto, showed a range of 92.8% to 94.5% for the certification of deaths. The lowest quality was observed in the indicators of deaths due to unspecified uterine cancer and cancer from poorly defined sites, mainly in Manizales and Bucaramanga. The analysis of principal components only identified a principal component shared by all the provinces, so a reduction in the dimensions could not be made (Fig. 2).

**Table 2:** Evaluation of the quality of the certification by cancer according to the place of occurrence of the death, Colombia, 2007-2011.

Geographic ordering ^**‡**^	Deaths from unspecified uterine cancer	Deaths from ill-defined cancer site	Deaths from cancer without age information	Deaths from cancer that were not certified by a physician	Duly certified deaths from cancer	Total deaths from cancer
Amazonas*	1.3	3.9	0.0	7.8	87.0	77
Antioquia*	0.7	6.4	0.0	0.0	92.9	27,411
Arauca*	1.6	4.3	0.0	0.2	93.9	507
Atlántico*	1.1	5.6	0.0	0.0	93.3	7,782
Bogotá D.C.**	0.6	5.8	0.0	0.0	93.6	33,332
Bolívar*	1.0	7.2	0.0	0.2	91.6	4,682
Boyacá*	1.0	6.2	0.0	0.1	92.7	4,024
Caldas*	0.9	6.7	0.0	0.0	92.4	1,876
Manizales¤	0.4	6.4	0.0	0.0	93.2	3,000
Caquetá*	1.8	8.4	0.0	0.3	89.5	998
Casanare*	2.0	9.2	0.0	0.0	88.8	609
Cauca*	0.9	5.7	0.0	4.0	89.4	3,387
Cesar*	1.1	6.9	0.0	0.0	92.0	2,383
Chocó*	2.0	7.5	0.0	2.0	88.4	441
Córdoba*	1.6	4.7	0.0	1.1	92.6	3,321
Cundinamarca*	1.3	6.3	0.0	0.0	92.4	5,713
Guainía*	0.0	0.0	0.0	0.0	100.0	15
Guajira*	2.6	4.8	0.0	1.6	91.0	765
Guaviare*	3.1	7.2	0.0	0.0	89.7	97
Huila*	0.5	6.8	0.0	0.2	92.5	4,010
Magdalena*	2.3	5.6	0.0	0.5	91.6	3,043
Meta*	0.9	6.4	0.0	0.0	92.7	2,957
Nariño*	1.0	4.8	0.0	0.7	93.5	2,105
Pasto¤	0.4	5.0	0.0	0.0	94.5	2,617
N. Santander*	1.3	6.5	0.0	0.1	92.1	4,881
Putumayo*	1.7	4.2	0.0	1.1	92.9	353
Quindío*	1.0	6.2	0.0	0.0	92.7	3,209
Risaralda*	0.7	6.9	0.0	0.0	92.4	4,946
San Andrés*	1.6	2.4	0.0	0.0	96.1	127
Santander*	1.3	7.1	0.0	1.1	90.5	1,947
Bucaramanga^†^	0.5	6.3	0.0	0.4	92.8	5,957
Sucre*	1.3	6.6	0.0	0.8	91.4	1,857
Tolima*	1.2	4.8	0.0	0.1	93.9	5,320
Valle*	1.5	6.3	0.0	0.1	92.2	6,546
Cali¤	0.5	5.9	0.0	0.0	93.7	14,205
Vaupés*	0.0	0.0	0.0	0.0	100.0	16
Vichada*	0.0	3.8	0.0	3.8	92.3	26
**Colombia**	0.9	6.1	0.0	0.2	92.8	164,542

‡ The geographic ordering of the country is defined in regions (two or more provinces), provinces

* (set of several municipalities), followed by special districts

**, municipalities¤ and metropolitan areas

† (two or more municipalities).

† Metropolitan Area of Bucaramanga (Bucaramanga, Floridablanca, Girón, Piedecuesta)

## Discussion

Colombia is among the countries with medium-high status according to the quality of certification of the cause of death ^13^. Although there is a broad framework for evaluating the quality of certification of general mortality, the components contemplated in this document do not differ from those considered for the period 2002-2006; and we included the classic indicators of quality for general mortality: proportions of deaths certified by a non-physician, without age, of unknown or nonspecific causes ^14^ ^-^ ^16^, and the indicators of cancer mortality quality: percentages of deaths due to poorly defined cancer site and deaths due to uterine cancer from non-specific sites ^17^ ^,^ ^18^.

This report confirms that Colombia is classified by the World Health Organization (WHO) -IARC as a class 2 country, with an average quality of mortality certification. According to this index, the countries classified as medium quality have a percentage of completeness that is between 70 and 90%; or have a percentage of completeness greater than 90% but with a percentage of deaths certified as signs, symptoms and ill-defined conditions that are between 10 and 20%; or have an *exhaustivity* (thoroughness) greater than 90%, with less than 10% of deaths certified as signs, symptoms and ill-defined conditions, but do not use codes of an international classification of diseases ^13^ ^,^ ^19^. It is possible that the classification of Colombia rises to that of a country with high quality in the certification of mortality since we have a percentage of deaths certified as signs, symptoms and ill-defined conditions that is less than 10%, and the last WHO’s coverage report of death reports that Colombia went from having coverage of 79.9% in the period 1990-1994 to 88.1% in 1995-1999, 93.1% in the period 2000-2004, and 98.5% in 2009 ^20^.

An improvement in all the certification quality indicators in Colombia was demonstrated, both globally and in the analysis disaggregated by provinces and some cities. The quality of the certification for general death was good with a percentage of deaths duly certified (93.7%), which improved the figure observed for the period 2002-2006 (92.8%). The results of the evaluation of the certification quality of cancer deaths (92.8%) also exceeded 91.5% that had been reported for this same period. Vaupés remained the province with the lowest indicators of the overall quality of certification, although its figures improved from 66.9% to 76.2%. The signs, symptoms and ill-defined conditions went from 1.5% to 2.0%.

Given the good performance of the quality indicators in these two aspects, it was found that mortality in Colombia as an input for the estimation of incident cases of cancer is valid, and it does not require adjustments for under-registration or correction for age, sex or undefined causes of death; then, in total for the period, it was only necessary to redistribute 101 deaths without sex information, 1,180 deaths without age information, 2,800 deaths not certified by physician and 19,937 deaths due to ill-defined causes among a total of 921,967 deaths without errors in certification.

For the specific case of cancer deaths, 1,452 deaths from ill-defined site uterine cancer (0.9% of total cancer deaths) should be redistributed according to standard methodologies; there would only remain 45 deaths by cancer without age (0.03%) to redistribute; 347 deaths by cancer not certified by physician (0.2%); and 9,986 deaths by cancer of ill-defined site (6.1%); of a total of 152,753 deaths from well-certified cancer.

In the cities which have a working population registry of cancer, the indicators were substantially better than in the rest of their provinces, and they were similar or exceeded the national average (Manizales 96.4%, Pasto 95.8% and Cali 96.4%), with the exception of Bucaramanga (92.3%). In the specific case of quality in the certification of cancer mortality, the indicators of the cities were also better than in the rest of their provinces (Manizales 93.2%, Pasto 94.5%, Bucaramanga 92.8%, Cali 93.7%), so it is considered that the information of general death and death from cancer in these areas is valid and serves as an input for the estimation of the mortality incidence ratio necessary for the estimation of incident cases of cancer. It should be noted that there is a higher proportion of deaths in these cities than in the rest of the municipalities of their respective provinces.

A recommendation for cities with cancer population registries would be that in the future, an analysis be performed in which the information about the diagnosis of cancer be crossed with the cause of death by cancer, in order to go deeper into the quality of the specific cause of cancer. However, this requires permission to cross databases with the identifier, which is not possible to perform at the present time. This particular issue is a call to the health authorities to allow these crossings with the different health related information registries.

This analysis was made in accordance with the site of occurrence of death; however, the analysis of mortality and the calculation of the mortality incidence ratio are made according to the place of residence of the deceased, so the quality of the certification described here may be slightly different from that observed in the analysis made in accordance with the place of habitual residence. 

*Phillips et al*, proposed a new methodology to establish the overall performance of the vital statistics system (SEV, for its acronym in Spanish) in each country, with the inclusion of six complementary dimensions with their respective indicators, a methodology that seeks to obtain reliable mortality information and monitor changes in time ^21^. In a second publication, *Phillips et al* defines the SEV performance index by ranges and for five categories (very low, <0.25, low, 0.25-0.49, medium, 0.50-0.69, high, 0.70-0.84, very high, ≥0.85). In this study, Colombia evaluated with the vital statistics information of 2008, presented an index of 82.5, with a high quality range ^22^. In future work, the application of these alternative estimation methods will be explored, according to the available information on mortality in Colombia.

## Conclusion

Certification quality indicators in Colombia improved for the studied period. Given the good performance of the quality indicators of both the certification of general death and cancer, it is considered that this is a valid input for the estimation of cancer incidence.
