# Supplementary material for: Quality of death certification in Colombia
Source: Colomb Med (Cali). 2018 Mar 30;49(1):121–7. doi: 10.25100/cm.v49i1.3155 (PMC6018815; doi:10.25100/cm.v49i1.3155)
Supplement: Related texts

# Research article: Calidad de la certificación de la muerte en Colombia

## Introducción

La medición y seguimiento de la calidad de la información de la mortalidad es un componente muy importante en la evaluación de los sistemas de información en salud de cada país ^1^. La mortalidad es un insumo fundamental para el análisis de situación en salud, monitorización de resultados de los programas de salud pública y planeación de necesidades en servicios de salud ^2^ y también es un insumo esencial para la estimación de los casos incidentes por cáncer ^3^^,^^4^. 

En Colombia, alrededor de 12% de la población está cubierta por un grupo de registros poblacionales de cáncer - RPC (Cali, Pasto, Bucaramanga, Manizales y Barranquilla) ^5^. La información de los RPC y los datos nacionales disponibles de mortalidad permiten hacer estimaciones de incidencia nacionales para cáncer. En un primer análisis el Instituto Nacional de Cancerología (INC**),** evaluó la calidad de la certificación de la mortalidad general y mortalidad por cáncer como parte del proceso de validación de las fuentes de información ^6^. En este reporte se revisa el avance de la calidad de la certificación de la mortalidad general y por cáncer con base en la información oficial del sistema nacional de registro de estadísticas vitales del Departamento Administrativo Nacional de Estadística (DANE). Esta información resulta necesaria para la estimación de incidencia por cáncer 2007- 2011 ^7^ y otros tipos de análisis con la mortalidad ^8^^-^^10^. 

La presente publicación presenta la evaluación de la calidad de la certificación de la muerte en Colombia en el periodo 2007-2011 según departamento de ocurrencia y además presenta los resultados para el distrito capital y las cuatro ciudades en las que funcionan los registros poblacionales de cáncer. 

## Materiales y Métodos

Se realizó un estudio descriptivo de la calidad de la información de mortalidad a partir de los certificados de defunción de Colombia. Los resultados se presentan desagregados según el departamento o ciudad en donde ocurrió la defunción. Para los departamentos que tengan municipios con RPC, se excluye los datos de estos municipios para evitar redundancia. Se incluyeron 32 departamentos, el distrito capital (Bogotá) y las ciudades de Manizales, Pasto, Cali y Bucaramanga (conformada por Bucaramanga, Floridablanca, Girón y Piedecuesta) que cuentan con registros poblacionales de cáncer activos y avalados por la Agencia Internacional para la Investigación en Cáncer (IARC, por sus siglas en inglés) ^11^, con un cubrimiento del 12% de la población que representa del país. La fuente de información es la base de datos oficial de la mortalidad del DANE para el quinquenio 2007-2011.

### Métodos estadísticos

La descripción de la calidad de la certificación se hizo a través de porcentajes simples. Cada aspecto relacionado con la falta de calidad en la información se consideró una sola vez; por ejemplo, si la defunción no era certificada por un médico, pero tenía además otro problema de certificación, este registro no se contaba dos veces, sino solo una vez como un registro que tenía fallas en la calidad de la certificación. Los resultados se presentan en un orden lógico de tal manera que los que aparecen en la primera columna de las tablas corresponden a la primera razón de falta de calidad; los que aparecen en la segunda columna corresponden a registros que tienen otras fallas diferentes a la primera y así de manera sucesiva (Tablas 1 y 2).

### Indicadores para la evaluación general de calidad de la información

#### La Clasificación Internacional de Enfermedades (CIE-10) se utilizó para la agrupación y codificación de las causas de muerte

Se construyeron los siguientes indicadores: las muertes por cáncer certificadas como de primario no establecido, de sitios mal especificados o como consecuencia de tumores metastásicos (C76-C80,C97); las muertes cardiovasculares de etiología mal definidas (I47.2, I49.0, I46, I50, I51.4, I51.5, I51.6, I51.9, I70.9); las lesiones de intencionalidad indeterminada (Y10-Y34,Y87.2); las muertes que fueron registradas como signos, síntomas y afecciones mal definidas (R00-R99); las muertes sin información de sexo; las muertes que no fueron certificadas por médicos; y las muertes que no tienen ninguna causa de error en la certificación.

### Indicadores en la evaluación de calidad de la información en la mortalidad por cáncer

Se construyeron los siguientes indicadores: las muertes por cáncer de útero de sitio no especificado (C55); las muertes por cáncer de primario no establecido, de sitios mal especificados o como consecuencia de un tumor metastásico sin un primario establecido (C76-C80, C97); las muertes por cáncer sin información de edad; las muertes por cáncer que no fueron certificadas por médico; y las muertes por cáncer bien certificadas.

### Plan de análisis

Con el fin de detectar una posible estructura de relación subyacente se realizó un análisis factorial de componentes principales, tanto para el análisis de la calidad de certificación de la mortalidad general como para el análisis de la certificación de la mortalidad por cáncer; se empleó el estadístico de Kaiser-Meyer-Olkin y la prueba de esfericidad de Bartlett con el fin de identificar si existía una estructura de relación subyacente ^12^. Aquellos factores que no se ajustaban con la solución propuesta de factores principales fueron excluidos del análisis; el número de factores se seleccionó con ayuda del análisis gráfico; en aquellos casos en los que se consideró apropiado, se hizo una rotación ortogonal utilizando el análisis de componentes principales como método de extracción y el varimax con normalización de Kaiser como método de rotación a partir del análisis gráfico. 

## Resultados 

En la evaluación de la calidad de la certificación de la muerte general para Colombia, se consideraron 984,159 defunciones para el quinquenio 2007-2011. Para la mortalidad general en el área de influencia de los registros poblacionales de cáncer de Bucaramanga, Cali, Manizales y Pasto se analizaron 138,716 defunciones (14.1% de la mortalidad nacional). 

### Indicadores de calidad de la certificación de muerte por causa general

El porcentaje nacional de muertes debidamente certificadas fue de un 93.7%, Los indicadores que más afectaron la calidad de la certificación fueron los signos, síntomas y afecciones mal definidas (2.0%) y las muertes cardiovasculares mal definidas (1.9%). Diez departamentos tuvieron un porcentaje de muertes sin errores en la certificación inferior al 90.1% y representaron únicamente el 6.6% de las muertes del país. Vaupés fue el departamento con más bajos indicadores en la certificación con 77.2%. La calidad de la certificación general por muertes sin errores estuvo en un rango de 92.3% a 96.4%, para las ciudades de Cali, Manizales, Bucaramanga y Pasto. La ciudad de Bucaramanga mostró una proporción menor en sus indicadores con respecto a las otras ciudades (Tabla 1).

**Tabla 1:** Evaluación general de la calidad de la certificación según lugar de ocurrencia de la defunción, Colombia, 2007-2011.

Ordenamiento geográfico^**‡**^	Muertes por cáncer sin primario establecido	Muertes cardiovasculares mal definidas	Lesiones de intencionalidad no determinada	Síntomas, signos y afecciones mal definidas	Muertes sin información de sexo	Muertes sin información de edad	Muertes que no fueron certificadas por un médico	Muertes sin errores en la certificación	Total muertes
Amazonas*	0.4	1.1	2.8	7.2	0.0	0.0	7.3	81.2	848
Antioquia*	1.2	1.4	0.9	1.5	0.0	0.0	0.0	94.9	148,653
Arauca*	0.5	1.7	1.1	1.7	0.0	0.0	0.3	94.7	4,494
Atlántico*	0.9	2.0	0.5	1.9	0.0	0.0	0.0	94.6	46,182
Bogotá D.C.**	1.2	1.4	1.6	2.7	0.0	0.0	0.0	93.1	159,432
Bolivar*	1.2	2.4	0.8	2.9	0.0	0.0	0.4	92.3	29,089
Boyacá*	0.8	3.1	1.5	1.4	0.0	0.0	0.3	92.8	29,704
Caldas*	1.0	2.2	0.8	1.2	0.1	0.0	0.1	94.7	12,166
Manizales§	1.2	1.1	0.4	0.9	0.0	0.0	0.0	96.4	15,651
Caquetá*	1.0	2.2	3.2	3.6	0.0	0.0	0.2	89.7	8,121
Casanare*	1.2	2.9	3.5	2.8	0.0	0.0	0.3	89.4	4,799
Cauca*	0.8	1.8	1.5	3.6	0.0	0.0	3.9	88.4	22,658
Cesar*	0.9	2.3	1.0	2.7	0.0	0.0	0.2	92.9	17,480
Chocó*	0.6	1.8	3.0	4.9	0.0	0.0	2.1	87.6	5,381
Córdoba*	0.6	2.4	0.7	2.1	0.0	0.0	0.8	93.3	26,801
Cundinamarca*	0.8	2.6	1.4	3.0	0.0	0.0	0.0	92.1	43,956
Guainía*	0.0	2.3	1.4	1.4	0.0	0.0	7.6	87.3	353
Guajira*	0.5	1.8	1.0	2.2	0.1	0.0	0.8	93.6	7,227
Guaviare*	0.5	1.3	3.4	3.3	0.4	0.0	0.1	91.0	1,334
Huila*	1.1	1.8	1.5	2.1	0.0	0.0	0.2	93.3	25,036
Magdalena*	0.8	2.3	0.7	2.1	0.0	0.0	0.4	93.6	21,732
Meta*	0.9	1.3	1.2	1.3	0.0	0.0	0.0	95.1	20,611
Nariño*	0.6	2.4	1.9	3.8	0.0	0.0	0.9	90,4	16,746
Pasto¤	0.9	1.6	0.9	0.8	0.0	0.0	0.0	95.8	14,671
N. Santander*	1.0	4.7	0.9	2.5	0.0	0.0	0.2	90.8	31,768
Putumayo*	0.4	1.2	4.7	2.1	0.1	0.0	1.7	89.8	3,572
Quindío*	1.2	1.4	0.2	0.4	0.0	0.0	0.0	96.7	16,577
Risaralda*	1.2	1.4	0.6	1.3	0.0	0.0	0.0	95.5	27,529
San Andrés*	0.3	2.5	0.2	0.9	0.0	0.0	0.1	95.9	952
Santander*	0.8	3.5	1.4	2.9	0.1	0.0	1.1	90.2	17,838
Bucaramanga^†^	1.2	2.4	0.4	3.2	0.0	0.0	0.4	92.3	30,725
Sucre*	0.9	3.1	0.6	1.7	0.0	0.0	0.5	93.2	13,360
Tolima*	0.7	1.3	0.7	0.9	0.0	0.0	0.2	96.2	35,803
Valle*	0.9	1.6	1.0	2.0	0.0	0.0	0.2	94.3	44,184
Cali¤	1.1	1.2	0.7	0.7	0.0	0.0	0.0	96.4	77,669
Vaupés*	0.0	0.8	3.8	3.2	0.0	0.0	15.0	77.2	474
Vichada*	0.2	2.4	2.9	3.1	0.7	0.0	1.0	89.7	583
**Colombia**	1.0	1.9	1.1	2.0	0.0	0.0	0.3	93.7	984,159

‡ El ordenamiento geográfico del país se encuentra definido en regiones (dos o más departamentos),

*departamentos (conjunto de varios municipios),

**seguido de distritos especiales,

§ municipios y

† áreas metropolitanas (dos o más municipios)

† Área metropolitana de Bucaramanga (Bucaramanga, Floridablanca, Girón, Piedecuesta)

El análisis de componentes principales mostró dos componentes que pueden explicar la falta de calidad: el primero está relacionado con la falta de certificación por un médico y la consecuente certificación inadecuada de la muerte ya sea como un signo, síntoma o afección mal definida o como una lesión de intencionalidad no determinada (Amazonas, Cauca, Guainía, Vaupés); el segundo componente tiene que ver con los departamentos en los que a pesar de tener certificación por médico hay errores en la certificación de las muertes por cáncer y de las muertes por causas cardiovasculares (Santander, Norte de Santander, Boyacá, Sucre) (Tabla 1 y Fig. 1).

**Figura 1:** Resultados del análisis de componentes principales en la evaluación general de la calidad de la certificación (gráfico rotado), Colombia, 2007-2011. Método de extracción: Análisis de componentes principales y Método de rotación: normalización Varimax con Kayser

### Indicadores de calidad de la certificación de muerte por cáncer

Se analizaron 164,542 defunciones. El porcentaje de muertes por cáncer debidamente certificadas fue 92.8%. Los indicadores que más afectaron la calidad de la certificación fueron las muertes por cáncer de sitio mal definido (6.1%) y las muertes por cáncer de útero de sitio mal definido (0.9%).

El análisis desagregado por departamentos mostró 10 departamentos con un porcentaje de muertes debidamente certificadas inferior al 91.6% que representaron el 9% del total de muertes por cáncer; Amazonas fue el departamento que tuvo más bajos indicadores en la calidad de certificación de mortalidad por cáncer (Tabla 2). En el análisis por ciudades, Bucaramanga, Cali, Manizales y Pasto, muestran un rango de 92.8% a 94.5% para la certificación de la muerte. La calidad más baja se observó en los indicadores de muertes por cáncer de útero sin especificar y por cáncer de sitio mal definido, principalmente en Manizales y Bucaramanga. El análisis de componentes principales solamente se identificó un componente principal que comparten todos los departamentos por lo que no se pudo hacer una reducción de las dimensiones (Fig. 2).

**Tabla 2:** Evaluación de la calidad de la certificación por cáncer según lugar de ocurrencia de la defunción, Colombia, 2007-2011.

Ordenamiento geográfico ^**‡**^	Muertes por cáncer de útero de sitio no especificado	Muertes por cáncer de sitio mal definido	Muertes por cáncer sin información de edad	Muertes por cáncer no certificadas por médico	Muertes por cáncer bien certificadas	Total muertes por cáncer
Amazonas*	1.3	3.9	0.0	7.8	87.0	77
Antioquia*	0.7	6.4	0.0	0.0	92.9	27,411
Arauca*	1.6	4.3	0.0	0.2	93.9	507
Atlántico*	1.1	5.6	0.0	0.0	93.3	7,782
Bogotá D.C.**	0.6	5.8	0.0	0.0	93.6	33,332
Bolívar*	1.0	7.2	0.0	0.2	91.6	4,682
Boyacá*	1.0	6.2	0.0	0.1	92.7	4,024
Caldas*	0.9	6.7	0.0	0.0	92.4	1,876
Manizales¤	0.4	6.4	0.0	0.0	93.2	3,000
Caquetá*	1.8	8.4	0.0	0.3	89.5	998
Casanare*	2.0	9.2	0.0	0.0	88.8	609
Cauca*	0.9	5.7	0.0	4.0	89.4	3,387
Cesar*	1.1	6.9	0.0	0.0	92.0	2,383
Chocó*	2.0	7.5	0.0	2.0	88.4	441
Córdoba*	1.6	4.7	0.0	1.1	92.6	3,321
Cundinamarca*	1.3	6.3	0.0	0.0	92.4	5,713
Guainía*	0.0	0.0	0.0	0.0	1,00.0	15
Guajira*	2.6	4.8	0.0	1.6	91.0	765
Guaviare*	3.1	7.2	0.0	0.0	89.7	97
Huila*	0.5	6.8	0.0	0.2	92.5	4,010
Magdalena*	2.3	5.6	0.0	0.5	91.6	3,043
Meta*	0.9	6.4	0.0	0.0	92.7	2,957
Nariño*	1.0	4.8	0.0	0.7	93.5	2,105
Pasto¤	0.4	5.0	0.0	0.0	94.5	2,617
N. Santander*	1.3	6.5	0.0	0.1	92.1	4,881
Putumayo*	1.7	4.2	0.0	1.1	92.9	353
Quindío*	1.0	6.2	0.0	0.0	92.7	3,209
Risaralda*	0.7	6.9	0.0	0.0	92.4	4,946
San Andrés*	1.6	2.4	0.0	0.0	96.1	127
Santander*	1.3	7.1	0.0	1.1	90.5	1,947
Bucaramanga^†^	0.5	6.3	0.0	0.4	92.8	5,957
Sucre*	1.3	6.6	0.0	0.8	91.4	1,857
Tolima*	1.2	4.8	0.0	0.1	93.9	5,320
Valle*	1.5	6.3	0.0	0.1	92.2	6,546
Cali¤	0.5	5.9	0.0	0.0	93.7	14,205
Vaupés*	0.0	0.0	0.0	0.0	100.0	16
Vichada*	0.0	3.8	0.0	3.8	92.3	26
**Colombia**	0.9	6.1	0.0	0.2	92.8	164,542

‡ El ordenamiento geográfico del país se encuentra definido en regiones

(dos o más departamentos),

*departamentos (conjunto de varios municipios),

**seguido de distritos especiales,

†municipios¤ y áreas metropolitanas (dos o más municipios).

† Área metropolitana de Bucaramanga (Bucaramanga, Floridablanca, Girón, Piedecuesta)

**Figura 2:** Resultados del análisis de componentes principales en la evaluación de la calidad de la certificación por cáncer (gráfico rotado), Colombia, 2007-2011. Método de extracción: Análisis de componentes principales y Método de rotación: normalización Varimax con Kayser

## Discusión

Colombia se encuentra entre los países con categoría media-alta de acuerdo con la calidad de certificación de la causa de muerte ^13^. Aunque existe un marco amplio para la evaluación de la calidad de certificación de la mortalidad general, los componentes contemplados en este documento no difieren de los considerados para el periodo 2002-2006 e incluyó los indicadores clásicos de calidad para la mortalidad general como son las proporciones de muertes certificadas por no médico, sin edad, de causas desconocidas o inespecíficas ^14^^-^^16^, y los indicadores de calidad de la mortalidad por cáncer: porcentajes de muertes por cáncer de sitio mal definido y muertes por cáncer de útero de sitio no especificado ^17^^,^^18^. 

Este reporte confirma que Colombia esté clasificado por Organización Mundial de la Salud (OMS) -IARC como un país de clase 2, con una calidad media en la certificación de mortalidad; de acuerdo con este índice, los países clasificados como de calidad media tienen un porcentaje de exhaustividad que está entre el 70 y el 90%, o tienen un porcentaje de exhaustividad mayor al 90% pero con un porcentaje de muertes certificadas como signos, síntomas y afecciones mal definidas que está entre el 10 y el 20%, o tienen una exhaustividad mayor al 90%, con menos de un 10% de las muertes certificadas como signos, síntomas y afecciones mal definidas, pero no usan códigos de una clasificación internacional de enfermedades ^13^^,^^19^. Es posible que la clasificación de Colombia suba a la de un país con calidad alta en la certificación de la mortalidad puesto que tenemos un porcentaje de muertes certificadas como signos, síntomas y afecciones mal definidas menor al 10%, y el último reporte de cobertura de la defunción de la OMS informa que Colombia pasó de tener una cobertura de 79.9% en el periodo 1990-1994 a 88.1% en 1995-1999, 93.1% en el periodo 2000-2004 y de un 98.5% para el 2009 ^20^. 

Se demostró una mejoría en todos los indicadores de calidad de certificación en Colombia, tanto globalmente, como en el análisis desagregado por departamentos y algunas ciudades. La calidad de la certificación por muerte general fue buena con un porcentaje de muertes debidamente certificadas (93.7%), lo cual mejoró la cifra observada para el periodo 2002-2006 (92.8%). Los resultados de la evaluación de la calidad de certificación de las muertes por cáncer (92.8%) también superaron el 91.5% que se había reportado para este mismo periodo. Vaupés siguió siendo el departamento con indicadores más bajos en la calidad general de la certificación, aunque sus cifras mejoraron desde un 66.9% a un 76.2%. Los signos, síntomas y afecciones mal definidas pasaron de 1.5% a 2.0%.

Ante el buen comportamiento de los indicadores de calidad, en estos dos aspectos, se encontró que la mortalidad en Colombia como insumo para la estimación de casos incidentes de cáncer es válida y no requiere ajustes por subregistro ni tampoco corrección por edad, sexo o causas mal definidas de defunción, pues, en total para el período solo habría que redistribuir 101 defunciones sin información de sexo, 1,180 defunciones sin información de edad, 2,800 defunciones no certificadas por médico y 19,937 defunciones por causas mal definidas entre un total de 921,967 defunciones sin errores en la certificación.

Para el caso específico de las muertes por cáncer se deben redistribuir de acuerdo con metodologías estándar 1,452 muertes por cáncer de útero de sitio mal definido (0.9% del total de muertes por cáncer); sólo quedaría por redistribuir 45 muertes por cáncer sin edad (0.03%), 347 muertes por cáncer no certificadas por médico (0.2%) y 9,986 muertes por cáncer de sitio mal definido (6.1%) entre un total de 152,753 muertes por cáncer bien certificadas.

En las ciudades en las que funciona un registro poblacional de cáncer, los indicadores resultaron ser sustancialmente mejores que en el resto del departamento y fueron similares o superaron al promedio nacional (Manizales 96.4%, Pasto 95.8% y Cali 96.4%) con excepción de Bucaramanga (92.3%). En el caso específico de la calidad en la certificación de la mortalidad por cáncer los indicadores de las ciudades también fueron mejores que en el resto del departamento (Manizales 93.2%, Pasto 94.5%, Bucaramanga 92.8%, Cali 93.7%) por lo que se considera que la información de muerte general y muerte por cáncer en estas áreas es válida y sirve como insumo para la estimación de la razón incidencia mortalidad necesaria para la estimación de casos incidentes de cáncer. Cabe destacar que existe una mayor proporción de muertes en estas ciudades que en el resto de municipios del departamento respectivo. 

Una recomendación para las ciudades con registros poblacionales de cáncer sería que a futuro se haga un análisis donde se cruce la información acerca del diagnóstico de cáncer con la causa de muerte por cáncer, con el fin de profundizar más en la calidad de la causa específica por cáncer. Sin embargo, para esto se requiere permiso de cruzar bases de datos con identificador, lo cual no es posible de realizar en la actualidad. Este tema en particular es un llamado a las autoridades en salud de permitir estos cruces con los distintos registros de información en salud relacionados.

Este análisis se hizo de acuerdo con el sitio de ocurrencia de la defunción; sin embargo, el análisis de mortalidad y el cálculo de la razón incidencia mortalidad se hacen según el sitio de residencia del fallecido, por lo que la calidad en la certificación que acá se describe puede ser ligeramente diferente a la observada en el análisis hecho de acuerdo con el sitio de residencia habitual.

*Phillips et al*, propusieron una nueva metodología para establecer el rendimiento global del sistema de estadísticas vitales (SEV) en cada país, con la inclusión de seis dimensiones complementarias con sus respectivos indicadores, metodología que busca obtener información confiable de mortalidad y monitorear los cambios en el tiempo ^21^. En una segunda publicación, *Phillips et al,* definen el índice de rendimiento del SEV por rangos y para cinco categorías (Muy bajo, <0.25; bajo, 0.25-0.49; medio, 0.50-0.69; alto, 0.70-0.84; muy alto, ≥0.85). En este estudio, Colombia evaluada con la información de estadísticas vitales de 2008 presentó un índice de 82.5 con un rango de calidad alto ^22^. En próximos trabajos se explorará la aplicación de estos métodos alternativos de estimación, según la información disponible de mortalidad en Colombia.

## Conclusión

Los indicadores de calidad de certificación en Colombia mejoraron para el periodo estudiado. Ante el buen comportamiento de los indicadores de calidad tanto de la certificación de la muerte general como por cáncer, se considera que ésta es un insumo válido para la estimación de incidencia de cáncer.
